# Novel Predictors and Risk Score of Treatment Failure in Peritoneal Dialysis-Related Peritonitis

**DOI:** 10.3389/fmed.2021.639744

**Published:** 2021-03-19

**Authors:** Xiang Liu, Aiya Qin, Huan Zhou, Xueqin He, Shamlin Cader, Sirui Wang, Yi Tang, Wei Qin

**Affiliations:** ^1^Division of Nephrology, Department of Medicine, West China Hospital, Sichuan University, Chengdu, China; ^2^West China School of Medicine, Sichuan University, Chengdu, China; ^3^Outpatient Department, West China Hospital, Sichuan University, Chengdu, China

**Keywords:** predictors, treatment failure, catheter removal, risk score, peritoneal dialysis associated peritonitis

## Abstract

**Objective:** Peritonitis is a severe complication in peritoneal dialysis (PD). This study was performed to identify predictors and establish a risk score for treatment failure in peritonitis patients.

**Methods:** A single-center, retrospective observational study was conducted. The basic demographic characteristics, clinical and laboratory data of all patients with peritonitis during the study period were documented and analyzed. Multivariate logistic regression was applied to examine independent predictors of treatment failure, and a risk prediction score was established.

**Results:** Three hundred fourteen episodes experienced by 241 patients were included in the final analysis. Logistic regression analysis indicated that PD duration (OR 1.017; P 0.005), fibrinogen (OR 1.327; P 0.021), high-density lipoprotein (OR 0.443; P 0.032), fungal infection (OR 63.413; *P* < 0.001), intestinal obstruction (OR 5.186, P 0.007), and diabetes mellitus (OR 2.451; P 0.018), hemodialysis history (OR 2.804, P 0.006) were independent predictors of treatment failure. The risk prediction score system showed a good calibration (*P* > 0.05) and discrimination (AUROC 0.80, *P* < 0.001).

**Conclusions:** Fibrinogen, PD duration, fungal infection, hemodialysis history, concurrent intestinal obstruction, or diabetes mellitus were independent risk factors for a poor peritonitis outcome, while the high-density lipoprotein was a protective factor. This novel risk prediction score system may be used to predict a high risk of treatment failure effectively.

## Introduction

Peritoneal dialysis (PD) is one of the main renal replacement treatments for patients with end-stage renal diseases (ESRD), accounting for about 11% of global dialysis patients (1). It provides a similar or better survival outcome vs, hemodialysis (HD) (2) and is more cost-effective. However, this therapy utilization has decreased recently in some countries due to shortage of high-quality evidence for prevention of peritonitis, poor control of dialysis center infections, and relatively high technique failure rate (3, 4).

PD-associated peritonitis (PDAP), as one of the most severe complications contributing to substantial morbidity and mortality of PD patients (5), was reported to be a significant cause of technical failure in PD patients (6), responsible for about 22% catheter removal, 18% transfer to HD, and 2–6% death (7). Furthermore, ongoing PDAP and an inadequate response to treatment can lead to extended hospitalization time, increased health care costs and damage on peritoneal structure and function (4, 8); once the treatment fails and the catheter is removed, only a small part of patients resume PD therapy (8). Despite the guidelines of PDAP, there are still considerably baffling variations in treatment outcomes of peritonitis in many centers and countries (4).

Recently the emphasis has also been put on improving peritonitis outcome and lowering the incidence of peritonitis. Early identification of risk factors predicting the poor outcome helps guide early treatment strategies and ameliorate prognosis. Although studies have reported the predictors of PDAP outcome previously, plenty of conflicting results were presented. PD duration (9), serum albumin (10), and concurrent DM (11) were observed to have prognostic value for peritonitis outcome in some studies, while some others thought these factors could not predict the treatment outcome (12). Therefore, further investigations are needed to explore these potential predictive factors.

In this study, risk factors of treatment failure of PDAP episodes were identified and turned into a novel risk score system, which may help predict poor outcome of PDAP and guide early interventions in these episodes.

## Materials and Methods

### Study Population

The single-center, retrospective observational study was carried out at the PD center of the West China Hospital, Sichuan University. Data regarding all peritonitis episodes from December 2014 to July 2018 were collected by reviewing case records. All patients received continuous ambulatory peritoneal dialysis (CAPD) using lactate-buffered glucose dialysis solution through Tenckhoff PD catheters with a twin-bag connection system (Baxter Healthcare, Guangzhou, China). The exclusion criteria included (1) patients with a previous kidney transplant; (2) episodes without bacterial cultures or missing data; (3) patients have received PD treatment for <1 month. All PD patients have accepted the established PD training curriculum conducted by nursing staff with appropriate qualifications and experience regularly. Patients were instructed to contact the hospital upon the appearance of cloudy effluent or digestive tract symptoms of unknown origin. When peritonitis was suspected, dialysate was collected for cell count and classification, gram staining and bacterial culture. Eight to ten milliliters of dialysate effluent were collected at the bedside into 2 (aerobic and anaerobic) blood-culture bottles, the culture would prolong to 5 days unless a positive signal was obtained within 48 h; another about 50 milliliters dialysate were gathered in a sterile tube for gram staining and blood agar culture after centrifugation of 3,500 rpm for 15 min, the culture of agar plates will last for 3 days under the aerobic and anaerobic circumstances unless organisms are detected.

This study was approved by the Medical Ethics Committee of West China Hospital of Sichuan University, Sichuan, China, on January 13, 2019, and written informed consent was obtained from each patient. This study was registered at the Thai Clinical Trials Registry (http://www.clinicaltrials.in.th/index.php?tp=regtrials&menu=trialsearch&smenu=fulltext&task=search&task2=view1&id=3339).

### Diagnosis

PDAP was diagnosed when at least two of the following manifestations were met: (1) abdominal pain and/or cloudy dialysate effluent; (2) dialysate effluent white cell count >100/ul or >0.1 × 10^9^/L with >50% polymorphonuclear cells, and (3) positive dialysate culture (5). Intestinal obstruction was considered if abdominal pain and/or distention, nausea/vomiting or cease of exhaust and defecation occurred and if it was combined with bowel dilation and extensive accumulation of gas and liquid (Gas-fluid levels in radiology).

### Treatment and Outcomes

The standard antibiotic protocol was used according to ISPD guidelines (5). Generally, initial therapy for PDAP should cover both gram-positive (G^+^) and gram-negative (G^−^) organisms, including first-generation cephalosporin or vancomycin combined with third-generation cephalosporin or aminoglycoside, and the regimen was modified according to the culture results and drug sensitivity.

Treatment success was defined as complete resolution of peritonitis (WBC count of <100/ul in the dialysate effluent with a relief of clinical manifestations) without the need for catheter removal; treatment failure included catheter removal or death. Catheter removal was considered in patients with refractory peritonitis (failure of the PD effluent to clear up after 5 days of appropriate antibiotic treatment), refractory exit-site or severe tunnel infection, or deterioration of the clinical condition as judged by the physician. Peritonitis-associated death was defined as death within 4 weeks of peritonitis, death with active peritonitis, or any death during hospitalization for a peritonitis episode (5).

### Basic Demographic, Clinical and Laboratory Data

Potential predictors were identified based on a comprehensive literature review and clinical experiences of nephrologists. These include basic demographic characteristics, such as gender, age, comorbidities, Charlson comorbidity index (13), etiology of ESRD, duration of PD, mean arterial pressure, history of antibiotic usage and history of HD; laboratory data including white blood cells (WBC), neutrophils, hemoglobin (Hb), serum albumin, creatinine (Cr), uric acid (UA), high-density lipoprotein (HDL) etc. that tested within 1 week of the diagnosis of peritonitis and causative organisms.

### Statistical Analysis

Statistical analysis was performed using IBM SPSS Statistics for Windows, Version 20.0 (IBM Corp, Armonk, NY, USA). All data were expressed as mean ± standard deviation (SD) for normally distributed data, median values with their interquartile range for skewed data and numbers (n) with percentage (%) for categorical variables. Data were analyzed by using the Chi-square test or Fisher's exact test for categorical variables, Student's *t*-test for normally distributed data and Wilcoxon rank sum test for continuous skewed variables. Variables whose *P* < 0.1 in the univariate analysis were selected for the final logistic regression to examine the independent risk factors of treatment failure. The stepwise procedure (forward: LR) was used to isolate the predictors. Odds ratios and 95% confidence intervals (CI) were calculated, and a two-tailed *P* < 0.05 was considered statistically significant. Data with <10% missingness were supplemented by the expectation-maximization (EM) method. Data with more than 20% of the values missingness were excluded from the final analysis.

A risk prediction score was converted for ease of application in clinical practice; To develop a simple integer-based point score for each variable, each β coefficient was divided by the model's minimum coefficient value and rounded up to the nearest integer to assign a score (14). To identify the cutoff values and evaluate the discrimination of the risk score, the area under the receiver operating characteristics curve (AUROC) was measured. Calibration was estimated by the Hosmer-Lemeshow goodness- of- fit test (15). To address the robustness of the risk score system, additional analysis was performed by excluding peritonitis episodes with: (1) age>65; (2) albumin>38 g/L;(3) culture negative; (4) intestinal obstruction; (5) subsequent peritonitis.

## Results

### Peritonitis Characteristics

**Figure 1:**
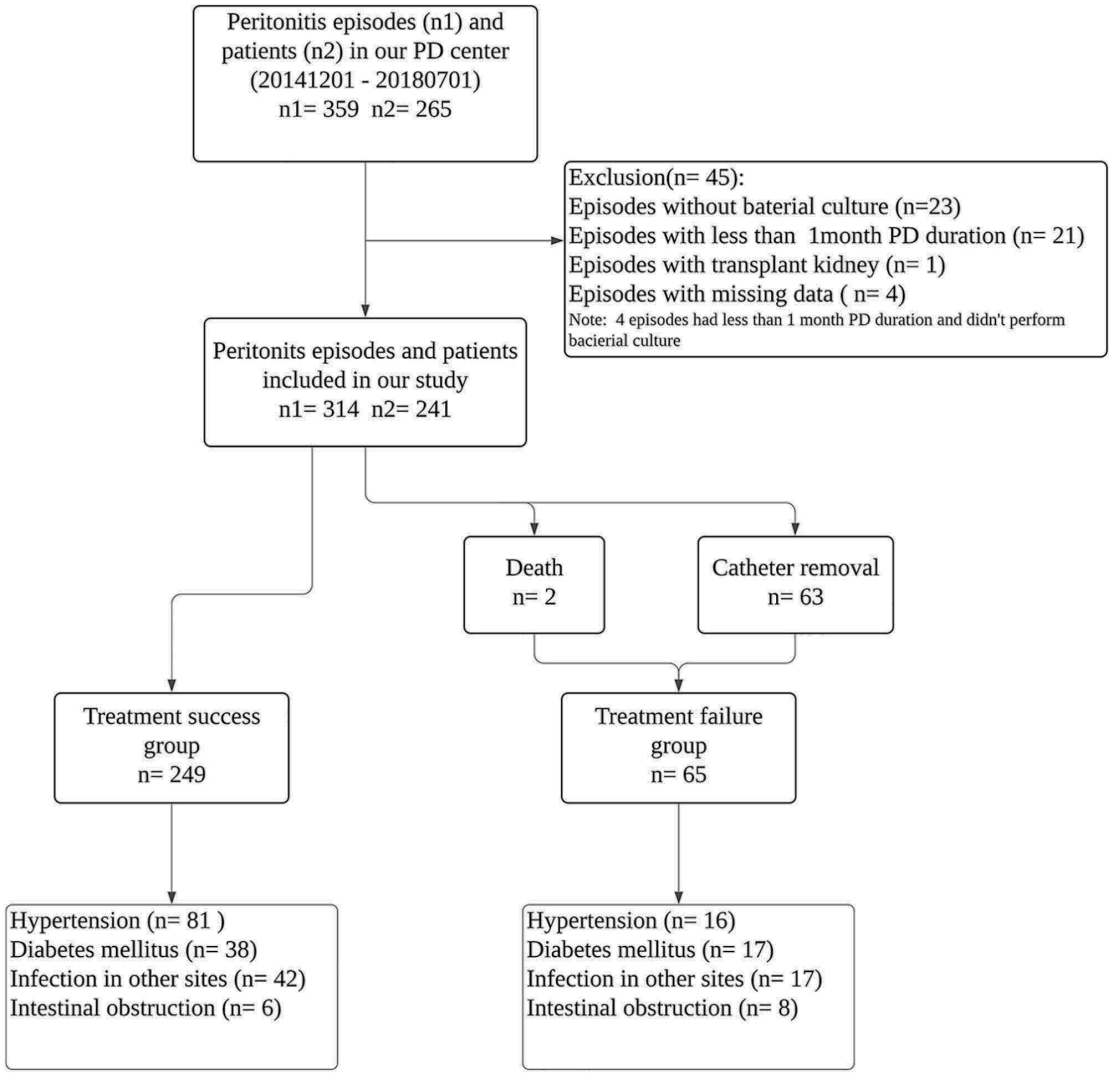
Flow chart for the study participants enrollment and outcomes.

There were 359 PDAP episodes in 265 patients during the study period. Twenty three episodes without bacteria culture, 21 episodes whose peritoneal dialysis duration was <1 month, one peritonitis with renal transplantation history and four episodes with missing data were excluded. Finally, a total of 314 episodes experienced by 241 patients were included in the final analysis (shown in Figure 1). The detailed data of patient baseline demographic characteristics are listed in Table 1, and causative PDAP organisms are listed in Table 2.

**Table 1:** The Basic demographic and clinical characteristics, laboratory data by treatment outcomes.

**Values**	**Total episodes (*n* = 314)**	**Success group (*n* = 249)**	**Failure group (*n* = 65)**	***P***
**Demographic and clinical characteristics**				
Male (*n*, %)	163 (51.9)	127 (51)	36 (55.4)	NS
Age at the onset of PD (year)	47.64 ± 13.42	50.49 ± 13.63	51.58 ± 13.41	NS
Age at the peritonitis (year)	50.56 ± 13.51	50.37 ± 13.67	51.29 ± 12.99	NS
PD duration (month)	28 (11–59)	25 (9–54)	39 (17.5–67.5)	0.003
Mean arterial pressure (MAP, mmHg)	102.69 ± 18.48	102.24 ± 18.4	103.74 ± 19.38	NS
History of antibiotics usage	89 (28.3)	71 (28.5)	18 (27.7)	NS
History of hemodialysis	57 (18.2)	40 (16.1)	17 (26.6%)	0.052
**Etiology of ESRD**				
Chronic glomerulonephritis	179 (57)	145 (58.2)	34 (52.3)	NS
Diabetic nephropathy	49 (15.6)	33 (13.3)	16 (24.6)	0.034
Immunologic nephropathy	15 (4.8)	12 (4.8)	3 (4.6)	NS
Others	71 (22.6)	59 (23.7)	12 (18.5)	NS
**Comorbidities (*****n*****, %)**				
Hypertension	97 (30.9)	81 (32.5)	16 (24.6)	NS
Diabetes mellitus	55 (17.5)	38 (15.3)	17 (26.2)	0.04
Infections in other sites	59 (18.8)	42 (16.9)	17 (26.2)	NS
Intestinal obstruction	14 (4.5)	6 (2.4)	8 (12.3)	0.002^a^
Charlson comorbidity index	3 (2–5)	3 (2–5)	4 (2–5)	NS
**Laboratory data**				
White blood cell (WBC, 10^9^/L)	6.88 (5.44–9.13)	6.75 (5.31–9.02)	7.58 (6.08–10.21)	0.034
Neutrophils (N, %)	0.77 (0.69–0.85)	0.76 (0.68–0.84)	0.8 (0.74–0.87)	0.005
Hemoglobin (Hb, g/L)	93.94 ± 19.63	95 ± 19.41	89.84 ± 20.11	NS
Albumin (Alb. g/L)	30.09 ± 6.02	30.62 ± 6.01	28.08 ± 5.63	0.002
Creatinine (Cr, umol/L)	778 (615.25–999.75)	773 (610–991)	823 (635.96–1056)	NS
Uric acid (UA, umol/L)	354.6 (304.63–412.93)	356 (309–412.95)	343 (290.6–423)	NS
Cholesterol (CHOL, mmol/L)	4.09 (3.48–4.81)	4.13 (3.55–4.92)	3.79 (3.33–4.42)	0.01
Triglyceride (TG, mmol/L)	1.33 (0.99–1.89)	1.3 (0.93–1.82)	1.49 (1.09–2.36)	0.024
High density lipoprotein (HDL, mmol/L)	1.15 ± 0.48	1.20 ± 0.49	0.95 ± 0.37	<0.001
Low density lipoprotein (LDL, mmol/L)	2.17 (1.67–2.85)	2.21 (1.75–2.89)	2.02 (1.43–2.38)	0.008
Fibrinogen (Fib, g/L)	5.25 ± 1.34	5.08 ± 1.30	5.89 ± 1.32	<0.001
Prothrombin time (PT, s)	12.22 (11.5–13)	12.2 (11.5–12.9)	12.52 (11.78–13.80)	0.021
Parathyroid hormone (PTH, pmol/L)	20.13 (9.64–39.52)	20.41 (10.6–40.23)	20.12 (6.96–36.30)	NS
Initial peritonitis (*n*, %)	208 (66.2)	173 (69.5)	35 (53.8)	0.018

*NS, not significant; ESRD, end stage of renal failure; eGFR, estimated glomerular filtrate rate*;

a *Fisher's exact test*.

**Table 2:** Causative organisms of the 314 peritonitis episodes.

**Causative organisms**	**Total episodes (*n* = 314)**	**Success group (*n* = 248)**	**Failure group (*n* = 55)**	***P***
**Culture negative** (*n*, %)^a^	173 (55.1)	149 (60.1)	24 (43.6)	0.026
**Gram positive**^a^ (*n*, %)	89 (28.3)	72 (29)	17 (30.9)	NS
*Coagulase-negative staphylococci*^b^	57 (64)	45 (62.5)	12 (70.6)	NS
*Streptococcus mitis*^b^	11 (12.4)	11 (15.3)	0 (0)	NS
*Staphylococcus aureus*^b^	9 (10.11)	6 (8.3)	3 (17.6)	NS^c^
*Enterococcus spp*	6 (6.7)	5 (6.9)	1 (5.9)	NS^c^
Others^b^	6 (6.7)	5 (6.9)	1 (5.9)	NS^c^
**Gram-negative** (*n*, %)^a^	37 (11.8)	25 (10.1)	12 (21.8)	0.016
*Escherichia coli*^b^	17 (45.9)	10 (40)	7 (58.3)	NS
*Pseudomonas aeruginosa*^b^	4 (10.8)	2 (8)	2 (16.7)	NS^c^
*Corynebacterium spp*^b^	3 (8.1)	3 (12)	0 (0)	NS^c^
*Klebsiella spp*^b^	2 (5.4)	2 (8)	0 (0)	NS^c^
Others^b^	11 (29.7)	8 (32)	3 (27.3)	NS^c^
**Polymicrobial**^a^	4 (1.3)	2 (0.8)	2 (3.6)	NS^c^
**Fungus**	11 (3.5)	1 (0.4)	10 (15.4)	<0.001^c^

*Fungus peritonitis was excluded when analyzing the other bacterial organisms*.

a *The number and percentage of causative organisms examined to all the episodes*.

b *The number and percentage of specific organism to related causative organisms examined*.

c *Fisher's exact test*.

### Clinical Features of PDAP Categorized by Treatment Outcomes

Among the 314 PDAP episodes included in the current study, treatment success was achieved in 249 (79.3%) episodes, and the remaining 65 (20.7%) episodes (two deaths and 63 catheter removals) resulted in treatment failure. Univariate analysis showed that variables associated with treatment failure included a higher level of peripheral WBC, neutrophils, TG, Fib, PT, and a lower level of Alb, CHOL, HDL, LDL (*P* < 0.05). Furthermore, patients with longer PD duration (*P* = 0.003), subsequent peritonitis (*P* = 0.018), or complicated with DM (*P* = 0.04) or intestinal obstruction (*P* = 0.002) were found to have a higher risk of treatment failure.

Details of the organisms responsible for the 314 peritonitis episodes are shown in Table 2. For causative organisms, 141 (44.9%) episodes were culture positive, of which 89 (28.3%) were due to G^+^ organisms, with *Coagulase negative staphylococcus* being the most common organism (64%), followed by *Streptococcus mitis* (12.4%) and *Staphylococcus aureus* (10.11%). 37 (11.8%) episodes were due to G^−^ organisms, of which *Escherichia coli* (45.9%) was the most common, followed by *Pseudomonas aeruginosa* (10.8%) and *Corynebacterium spp* (8.1%); 4 (1.3); and 11 (3.5%) peritonitis episodes infected multiple bacteria and fungus, respectively. The remaining 173 (55.1%) episodes are negative cultures.

To eliminate the interference of fungus for its high rate of poor outcomes (5), fungal peritonitis was excluded when we analyzed the effect of other bacteria on treatment outcome. Culture-negative peritonitis was found to have a significantly lower catheter removal rate than in culture-positive episodes (*p* = 0.026). A significant difference was observed in the percentages of G^−^ bacteria between treatment failure and success groups (*P* = 0.016), while the differences were not seen in episodes caused by G^+^ bacteria. Among gram-positive and gram-negative organisms, no single bacteria was found to have an association with treatment failure.

### The Predictors of Treatment Failure in PDAP

**Figure 2:**
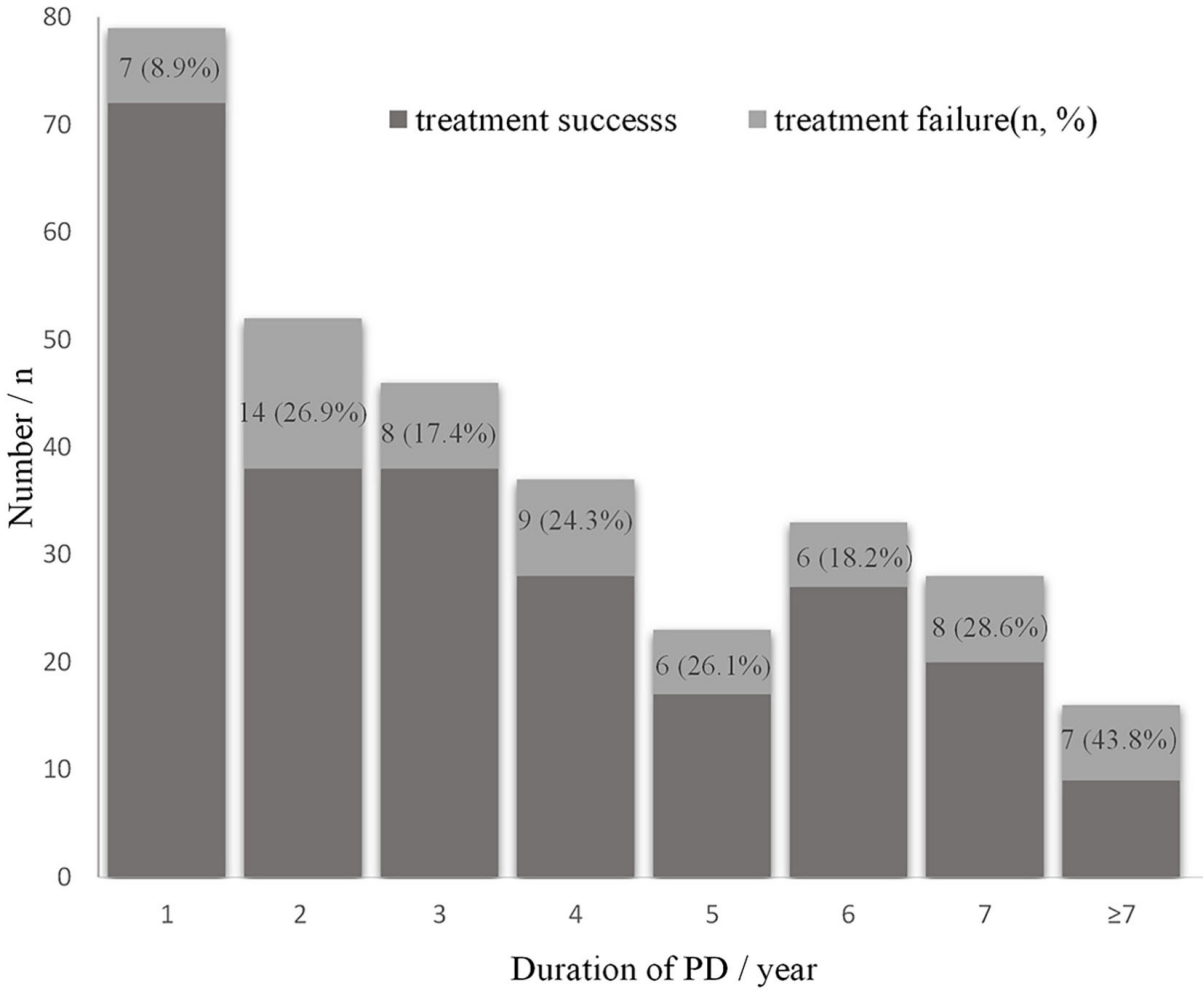
Distribution of treatment outcomes by peritoneal dialysis time.

Logistic regression suggested that HDL, Fibrinogen, PD duration, fungal infection, intestinal obstruction, DM, HD history were demonstrated to be independent predictors of treatment failure in peritonitis (Table 3). Specifically, the odds of treatment failure increased to 1.327 with every 1 g/L Fib increment (*P* = 0.021), 5.186, 2.451 and 2.804 in patients with intestinal obstruction (*P* = 0.007), DM (*P* = 0.018) and history of HD (*P* = 0.006), respectively, compared to those without these complications. In terms of the relationship between PD duration and treatment outcomes, the distribution of treatment outcomes of the 314 peritonitis episodes by dialysis duration was shown in Figure 2. The proportion of catheter removal or death rose over the PD time. In the regression model, the risk of treatment failure increased to 1.017 with every 1 month longer (95%CI 1.005 to 1.028; *P* = 0.005) (Table 3).

**Table 3:** Multivariate logistic regression model on prediction of the peritonitis treatment failure.

**Values**	***P***	**OR**	**95% CI**
High density lipoprotein	0.032	0.443	0.211–0.993
Fibrinogen	0.021	1.327	1.043–1.687
PD duration/year	0.005	1.017	1.005–1.028
Intestinal obstruction	0.007	5.186	1.575–17.069
Diabetes mellitus	0.018	2.451	1.167–5.148
Fungal peritonitis	<0.001	63.413	7.421–541.845
Hemodialysis history	0.006	2.804	1.342–5.860

*OR, odds ratio; CI, confidence intervals*.

### Risk Score

**Figure 3:**
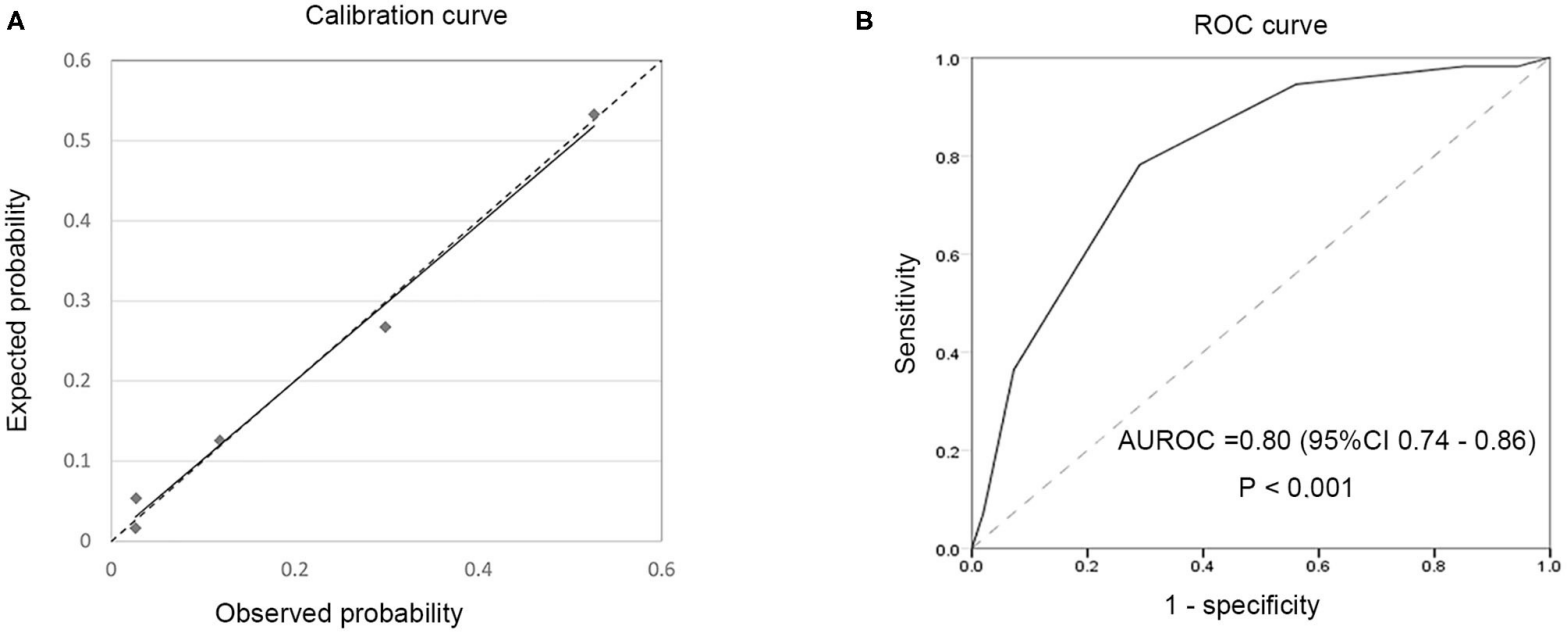
Calibration curve for expected vs. observed probability in the overall cohort **(A)** and receiver operating curve of risk predicting model in PDAP episodes **(B)**.

Episodes of fungus peritonitis (*n* = 11) were excluded in the final risk score system because of the high rate of catheter removal and it is an indication of catheter removal in ISPD guideline. Table 4 shows the β regression coefficient and the risk score of each predictor, the total score ranges from 0 to 8 points. The expected probability of treatment failure calibrated well with the observed probability (*P* > 0.05, shown in Figure 3A); the area under the receiver operating characteristic of the 303 episodes is 0.80 (95% CI 0.74–0.86, *P* < 0.0001) (shown in Figure 3B), >0.7, showing a good discrimination capability. The optimal cutoff value for risk score was 3.5 points (sensitivity 78%, specificity 71%, Youden's index 0.491), indicating that a patient with a score of 4 or more had a high risk of treatment failure when experiencing a PDAP episode.

**Table 4:** ß coefficient and corresponding risk score developed from risk prediction model.

**Risk factors**	**Adjusted OR (95% CI)**	***P*-value**	**ß-coefficients**	**Transformed score**	**Assigned score**
HDL ≤ 1.5mmol/L					
No	1.0 (Reference)	-	-	0	0
Yes	4.78 (1.35–16.97)	0.015	1.565	1.79	2
Fibrinogen ≥ 5.25 g/L					
No	1.0 (Reference)	-	-	0	0
Yes	2.83 (1.39–5.79)	0.004	1.042	1.19	1
Diabetes mellitus					
No	1.0 (Reference)	-	-	0	0
Yes	2.40 (1.13–5.12)	0.024	0.875	1	1
Intestinal obstruction					
No	1.0 (Reference)	-	-	0	0
Yes	6.05 (1.76–20.74)	0.004	1.800	2.06	2
Hemodialysis history					
No	1.0 (Reference)	-	-	0	0
Yes	2.88 (1.37–6.05)	0.005	1.057	1.21	1
PD duration ≥ 32 month					
No	1.0 (Reference)	-	-	0	0
Yes	2.51 (1.23–5.14)	0.011	0.922	1.05	1

*HDL, High density lipoprotein. Fungal peritonitis were excluded*.

### Subgroup and Sensitivity Analysis

Supplementary Table 1 shows the characteristics of episodes complicated with ileus, which revealed that patients with a higher level of fibrinogen, neutrophils, and those infected by gram-negative organisms, especially the *E. coli*., taking up 88.9%, had a higher possibility of concurrent intestinal obstruction.

Logistic regression analysis was performed again after exclusion of fungal peritonitis and subsequent peritonitis, the results were comparable (Supplementary Table 2), indicating the good robustness of these predictors.

For risk score system, stratification of age, albumin, and exclusion of culture-negative, intestinal obstruction did not change the prediction model performances, the discrimination ranged from 0.74 to 0.80, and all *P*-values of Hosmer-Lemeshow statistic were >0.05 (Supplementary Table 3), indicating the good robustness of the risk score system.

## Discussion

In this retrospective study of 314 PDAP episodes in 3.6 years, we found that high fibrinogen level, long PD duration, fungal peritonitis, HD history, intestinal obstruction or DM were independent predictors of a poor treatment outcome. In contrast, HDL seemed to be a protective factor in these patients. To facilitate the clinical application, we developed a novel risk score system based on our findings and found that PDAP patients with risk score of four or more were in high risk of treatment failure. Sensitivity analysis indicated good robustness of these predictors and a risk score system.

Our study provides new implications and directions in predicting adverse outcomes in peritonitis; these novel predictors may be incorporated into a large prediction model (15). All these risk factors are easily obtained with low cost from medical history records and routine biochemical examination to be of practical clinical value. According to the risk stratification, physicians may estimate the severity of peritonitis early, and then give the appropriate care and treatment strategy. For patients with a score ≥4, more comprehensive testing and assessment, more closely monitor, timely adjustments of antibiotics, and early nephrology care as well as other expert health care services should be considered.

The association of HD history and prognosis of peritonitis in PD patients has has never been detected in previous researches. Some patients in our center have HD history for some reasons; some begin HD unaware of PD as a treatment choice for ESRD, some transfer to PD under the consideration of the financial situation, living condition or HD complications etc. In the current study, the HD history is demonstrated to be a marked predictor of technical failure in peritonitis episodes (*P* = 0.006, 0R 2.804). No studies to date have assessed the possible drivers, but a higher rate of technique failure is also found in patients who transfer from HD to PD in several studies (16, 17). These patients may have difficulties in adapting the manual management of PD after getting used to HD; this may be a contributor. Furthermore, psychosocial factors may also affect; more psychosocial supports than other patients when starting dialysis at home are needed, given that acute dialysis initiation could raise feelings of anxiety and reduce the patient's confidence in home therapy (17).

Previously, albumin was detected to be a predictor of treatment failure in PDAP (18), partly because of malnutrition; the albumin level was associated with technique failure in the univariate analysis in this study, but not a predictor in the multivariate logistic regression analysis. Controversial results have been reported about the effect of DM on PDAP outcome. Some studies revealed that DM (9, 10) could not predict the peritonitis outcome. However, Krishnan et al. (9) excluded peritonitis with culture-negative infection, and the indication of catheter loss in a study conducted by Yang CY was not clear (10). In the present analysis, DM is recognized as a risk factor of treatment failure of PDAP, similar to previous research (11). It was reported that DM could impair peritoneal defense (19), which may adversely affect the prognosis of PDAP; in addition, as long-term exposure to glucose dialysate may cause dysfunction of the peritoneum, which impairs the peritoneal immunologic reactions against bacteria and increases the severity of peritonitis (20); furthermore, some patients with DM are complicated with diabetic retinopathy, PD needs to manage manually at home; this probably results in operation errors, which may be a contributor to technique failure. Longer PD duration was identified as a predictor of worse clinical outcome of PDAP (*P* = 0.005), in line with a previous study (9). Our results underscore the importance of timely management and meticulous care of peritonitis in patients with DM or long PD duration for favorable prognosis.

Notably, fibrinogen concentration was recognized as a predictor of treatment failure in peritonitis patients in our cohort. Although fibrin deposition may ameliorate systematic inflammation by preventing bacterial spread (21), fibrin mesh formed from extensive exudation and deposition of fibrin can facilitate bacteria's proliferation and prevent them from host phagocytic cells attack and bactericidal effects of antibiotics (22, 23). Additionally, fibrin exudation and deposition may lead to fibrous capsulation and paralytic intestinal obstruction (24), as our study observed, PDAP patients with intestinal obstruction seemed to have increased Fib level (Supplementary Table 1); Intestinal obstruction has been proved to be linked with technical failure in fungal (25) and bacterial PDAP (26) previously. Our results further confirmed these findings. For one reason, ileus could result in bowel immobilization and dysfunction of nutrients absorption; for another, ileus has a higher possibility of infections with gram-negative organisms, especially *E. coli* (Supplementary Table 1), which may lead to more severe peritonitis compared to other organisms (27). The exact mechanism needs to be studied further.

We also found that patients in the treatment failure group are characterized as lower HDL concentrations. As far as we know, this is the first study to reveal the effect of dyslipidemia on the peritonitis treatment outcome. HDL can exert antioxidant and anti-inflammatory activity by inhibiting oxidative stress and the formation of pro-inflammatory oxidized lipids; meanwhile, HDL can attenuate systematic inflammation by increased scavenging of endotoxin (28), this anti-inflammatory and antioxidant effect of HDL may play a crucially positive role in the prognosis of peritonitis. Notably, HDL in ESRD patients has been reported to have impaired antioxidant and anti-inflammatory effect and has even a pro-oxidant and pro-inflammatory effect because of functional and structural abnormalities (28, 29). Therefore, worse outcomes in dialysis patients were reported to be associated with HDL increment (30). There are many controversies, and uncertainties in the relationship between lipid disorders and peritoneal dialysis, the mechanisms of the effects of dyslipidemia on the prognosis of peritonitis require further investigation.

This study has several limitations. Firstly, this is a retrospective, single-center research, which may lead to bias in our findings. Secondly, considering that the external validation is not performed, the generalization of risk prediction score was restricted. Also, negative culture rate (55.1%) is high compared to the 15% rate suggested by the IPSD (5). It might be due to the use of antibiotics before sample collection. Many patients in our center live in villages or cities far away from our hospital, antibiotics have been prescribed to them by local doctors; furthermore, even though the culture protocol conforms to the IPSD guideline recommendations (5), methods that could improve the rate of culture-positive such as usage of rapid blood-culture bottle kits or lysis centrifugation technique should be considered. Nevertheless, the distribution of causative organisms in our study is following previously reported studies, and the empiric therapy has satisfactory outcomes with 80% rate of treatment success; besides, our findings are independent of the type of organisms, demonstrating that these findings can apply to some PD cohorts elsewhere. Finally, all patients in our study are Chinese and perform CAPD; the generalizing these findings may be limited to Asian and PD patients with CAPD modality. Even though all patients in our center take CAPD for financial considerations rather than automated peritoneal dialysis (APD), which is more commonly used in developed countries, such as the US (31), no significant differences are found in technique survival between CAPD and APD as reported by a few large representative cohort studies (31–34).

In conclusion, high Fib level, long PD duration, fungal infection, HD history, concurrent intestinal obstruction or DM were found to be independent predictors of treatment failure of PDAP, while HDL seems to be a protective factor in patients with peritonitis. A novel risk score system could be employed to predict the risk of treatment failure of PDAP easily.

## Data Availability Statement

The raw data supporting the conclusions of this article will be made available by the authors, without undue reservation.

## Author Contributions

XL, AQ, YT, and WQ: conception and design. XL, AQ, HZ, XH, SC, and SW: administrative support. XL, AQ, HZ, YT, and WQ: collection and assembly of data. XL, AQ, HZ, XH, SC, SW, YT, and WQ: data analysis and interpretation. XL, AQ, HZ, XH, SC, YT, and WQ: manuscript writing. XL, AQ, HZ, XH, SC, SW, YT, and WQ: final approval of manuscript.

## Conflict of Interest

The authors declare that the research was conducted in the absence of any commercial or financial relationships that could be construed as a potential conflict of interest.

## References

[1] Jain AK Blake P Cordy P Garg AX. Global trends in rates of peritoneal dialysis. J Am Soc Nephrol. (2012) 23:533–44. 10.1681/ASN.2011060607 22302194 PMC3294313

[2] Wong B Ravani P Oliver MJ Holroyd-Leduc J Venturato L Garg AX. Comparison of patient survival between hemodialysis and peritoneal dialysis among patients eligible for both modalities. Am J Kidney Dis. (2018) 71:344–51. 10.1053/j.ajkd.2017.08.028 29174322

[3] Li PKT Chow KM Van de Luijtgaarden MWM Johnson DW Jager KJ Mehrotra R. Changes in the worldwide epidemiology of peritoneal dialysis. Nat Rev Nephrol. (2017) 13:90–103. 10.1038/nrneph.2016.181 28029154

[4] Cho Y Johnson DW. Peritoneal dialysis-related peritonitis: towards improving evidence, practices, and outcomes. Am J Kidney Dis. (2014) 64:278–89. 10.1053/j.ajkd.2014.02.025 24751170

[5] Li PK Szeto CC Piraino B De Arteaga J Fan S Figueiredo AE. ISPD peritonitis recommendations: 2016 update on prevention and treatment. Perit Dial Int. (2016) 36:481–508. 10.3747/pdi.2016.00078 27282851 PMC5033625

[6] Hsieh YP Chang CC Wang SC Wen YK Chiu PF Yang Y. Predictors for and impact of high peritonitis rate in Taiwanese continuous ambulatory peritoneal dialysis patients. Int Urol Nephrol. (2015) 47:183–9. 10.1007/s11255-014-0763-5 25034275

[7] Mehrotra R Devuyst O Davies SJ Johnson DW. The current state of peritoneal dialysis. J Am Soc Nephrol. (2016) 27:3238–52. 10.1681/ASN.2016010112 27339663 PMC5084899

[8] Choi P Nemati E Banerjee A Preston E Levy J Brown E. Peritoneal dialysis catheter removal for acute peritonitis: a retrospective analysis of factors associated with catheter removal and prolonged postoperative hospitalization. Am J Kidney Dis. (2004) 43:103–11. 10.1053/j.ajkd.2003.08.046 14712433

[9] Krishnan M Thodis E Ikonomopoulos D Vidgen E Chu M Bargman JM. Predictors of outcome following bacterial peritonitis in peritoneal dialysis. Perit Dial Int. (2002) 22:573–81. 10.1177/089686080202200508 12455568

[10] Yang CY Chen TW Lin YP Lin CC Ng YY Yang WC. Determinants of catheter loss following continuous ambulatory peritoneal dialysis peritonitis. Perit Dial Int. (2008) 28:361–70. 10.1177/089686080802800410 18556378

[11] Tsai C-C Lee J-J Liu T-P Ko W-C Wu C-J Pan C-F. Effects of age and diabetes mellitus on clinical outcomes in patients with peritoneal dialysis-related peritonitis. Surg Infect. (2013) 14:540–6. 10.1089/sur.2012.195 24116738

[12] Kofteridis DP Valachis A Perakis K Maraki S Daphnis E Samonis G. Peritoneal dialysis-associated peritonitis: clinical features and predictors of outcome. Int J Infect Dis. (2010) 14:e489–93. 10.1016/j.ijid.2009.07.016 19926324

[13] Charlson M Szatrowski TP Peterson J Gold J. Validation of a combined comorbidity index. J Clin Epidemiol. (1994) 47:1245–51. 10.1016/0895-4356(94)90129-5 7722560

[14] Moons KGM Harrell FE Steyerberg EW. Should scoring rules be based on odds ratios or regression coefficients? J Clin Epidemiol. (2002) 55:1054–5. 10.1016/S0895-4356(02)00453-5 12464384

[15] Nochaiwong S Ruengorn C Koyratkoson K Thavorn K Awiphan R Chaisai C. A clinical risk prediction tool for peritonitis-associated treatment failure in peritoneal dialysis patients. Sci Reports. (2018) 8:14797. 10.1038/s41598-018-33196-2 30287920 PMC6172229

[16] Lobbedez T Verger C Ryckelynck J-P Fabre E Evans D. Outcome of the sub-optimal dialysis starter on peritoneal dialysis. Report from the French Language Peritoneal Dialysis Registry (RDPLF). Nephrol Dial Transplantation. (2013) 28:1276–83. 10.1093/ndt/gft018 23476042

[17] Lobbedez T Verger C Ryckelynck JP Fabre E Evans D. Is assisted peritoneal dialysis associated with technique survival when competing events are considered? Clin J Am Soc Nephrol. (2012) 7:612–8. 10.2215/CJN.10161011 22344506

[18] Tian Y Xie X Xiang S Yang X Lin J Zhang X. Risk factors and outcomes of early-onset peritonitis in Chinese peritoneal dialysis patients. Kidney Blood Pressure Res. (2017) 42:1266–76. 10.1159/000485930 29248923

[19] Santamaría B Sanz A Justo P Catalán M Sánchez-Niño MD Benito A. Peritoneal defence–lessons learned which apply to diabetes complications. Nephrol Dial Transplantation. (2006) 21(Suppl. 2):ii12–5. 10.1093/ndt/gfl185 16825252

[20] Wu J Yang X Zhang YF Wang YN Liu M Dong XQ. Glucose-based peritoneal dialysis fluids downregulate toll-like receptors and trigger hyporesponsiveness to pathogen-associated molecular patterns in human peritoneal mesothelial cells. Clin Vaccine Immunol. (2010) 17:757–63. 10.1128/CVI.00453-09 20200188 PMC2863392

[21] Qiu G Gribbin E Harrison K Sinha N Yin K. Inhibition of gamma interferon decreases bacterial load in peritonitis by accelerating peritoneal fibrin deposition and tissue repair. Infect Immunity. (2003) 71:2766–74. 10.1128/IAI.71.5.2766-2774.2003 12704151 PMC153258

[22] Dunn DL Simmons RL. Fibrin in peritonitis. III. The mechanism of bacterial trapping by polymerizing fibrin. Surgery. (1982) 92:513–9. 7051387

[23] Rotstein OD Pruett TL Simmons RL. Fibrin in peritonitis. V. Fibrin inhibits phagocytic killing of *Escherichia coli* by human polymorphonuclear leukocytes. Annals Surg. (1986) 203:413–9. 10.1097/00000658-198604000-00013 3516086 PMC1251127

[24] Mizuno M Ito Y Mizuno T Harris CL Suzuki Y Okada N. Membrane complement regulators protect against fibrin exudation increases in a severe peritoneal inflammation model in rats. Am J Physiol Renal Physiol. (2012) 302:F1245–51. 10.1152/ajprenal.00652.2011 22338087

[25] Lo SHK Chan Ck Shum Hp Chow VCC Mo Kl Wong KS. Risk factors for poor outcome of fungal peritonitis in Chinese patients on continuous ambulatory peritoneal dialysis. Perit Dial Int. (2003) 23(Suppl. 2):S123–6. 10.1177/089686080302302s25 17986530

[26] Ram R Swarnalatha G Rao CSS Naidu GD Sriram S Dakshinamurty KV. Risk factors that determine removal of the catheter in bacterial peritonitis in peritoneal dialysis. Perit Dial Int. (2014) 34:239–43. 10.3747/pdi.2012.00343 24676745 PMC3968112

[27] Szeto CC Chow VCY Chow KM Lai RWM Chung KY Leung CB. Enterobacteriaceae peritonitis complicating peritoneal dialysis: a review of 210 consecutive cases. Kidney Int. (2006) 69:1245–52. 10.1038/sj.ki.5000037 16467787

[28] Vaziri ND. HDL abnormalities in nephrotic syndrome and chronic kidney disease. Nat Rev Nephrol. (2016) 12:37–47. 10.1038/nrneph.2015.180 26568191

[29] Moradi H Vaziri ND Kashyap ML Said HM Kalantar-Zadeh K. Role of HDL dysfunction in end-stage renal disease: a double-edged sword. J Ren Nutr. (2013) 23:203–6. 10.1053/j.jrn.2013.01.022 23611547 PMC3664234

[30] Chang TI Streja E Soohoo M Ko GJ Rhee CM Kovesdy CP. Increments in serum high-density lipoprotein cholesterol over time are not associated with improved outcomes in incident hemodialysis patients. J Clin Lipidol. (2018) 12:488–97. 10.1016/j.jacl.2018.01.010 29456130

[31] Balasubramanian G McKitty K Fan SLS. Comparing automated peritoneal dialysis with continuous ambulatory peritoneal dialysis: survival and quality of life differences? Nephrol Dial Transplantation. (2011) 26:1702–8. 10.1093/ndt/gfq607 20921296

[32] Mehrotra R Chiu Y-W Kalantar-Zadeh K Vonesh E. The outcomes of continuous ambulatory and automated peritoneal dialysis are similar. Kidney Int. (2009) 76:97–107. 10.1038/ki.2009.94 19340090

[33] Badve SV Hawley CM McDonald SP Mudge DW Rosman JB Brown FG. Automated and continuous ambulatory peritoneal dialysis have similar outcomes. Kidney Int. (2008) 73:480–8. 10.1038/sj.ki.5002705 18046315

[34] Johnson DW Hawley CM McDonald SP Brown FG Rosman JB Wiggins KJ. Superior survival of high transporters treated with automated versus continuous ambulatory peritoneal dialysis. Nephrol Dial Transplantation. (2010) 25:1973–9. 10.1093/ndt/gfp780 20097847

