# Supplementary material for: Novel Predictors and Risk Score of Treatment Failure in Peritoneal Dialysis-Related Peritonitis
Source: Front Med (Lausanne). 2021 Mar 19;8:639744. doi: 10.3389/fmed.2021.639744 (PMC8033636; doi:10.3389/fmed.2021.639744)
Supplement: Supplementary file 1 [file Data_Sheet_1.DOCX]

**Appendices**

**Supplemental Table 1. Characteristics of episodes with intestinal obstruction.**

| variables | No obstruction group（n=300） | obstruction group (n=14) | P |
| --- | --- | --- | --- |
| Male (n, %) | 127(51) | 36(55.4) | NS |
| PD duration(month) | 29（11-59） | 23（16.75-47.75） | NS |
| White blood cell (WBC, 10^9^/L) | 6.85(5.42 – 9.00) | 9.62(6.48 – 12.13) | NS |
| Neutrophils (N, %) | 0.76±0.10 | 0.85±0.07 | 0.001 |
| Albumin (Alb, g/L) | 30.12± 6.02 | 29.34±6.22 | NS |
| Cholesterol (mmol/L) | 4.10 (3.48 – 4.85) | 3.87 (3.26 – 4.41) | NS |
| Triglyceride (mmol/L) | 1.33 (0.97 – 1.86) | 1.80 (1.09 – 2.83) | NS |
| High density lipoprotein (mmol/L) | 1.16±0.48 | 0.94 ± 0.41 | NS |
| Low density lipoprotein (mmol/L) | 2.27±0.85 | 1.99± 0.76 | NS |
| Fibrinogen (mmol/L) | 5.21± 1.34 | 6.02± 1.31 | 0.027 |
| Prothrombin time (s) | 12.2 (11.5 – 13) | 12.95 (11.88 -14.15) | NS |
| Parathyroid hormone (pmol/L) | 19.8 (8.78 – 39.68) | 22.47(20.01 - 45.49) | NS |
| Causative organisms (n, %) |  |  |  |
| Culture negative | 171(57) | 2(14.3) | 0.002 |
| Gram positive | 88(29.3) | 1(7.1) | NS ^a^ |
| Gram negative | 28(9.3) | 9(64.3) | <0.001 ^a^ |
| Polymicrobial | 3(1) | 1(7.1) | NS ^a^ |
| Fungus | 10(3.3) | 1(7.1) | NS ^a^ |

^a^ Fisher’s exact test. PD: peritoneal dialysis.

**Supplemental Table 2. Multivariate logistic regression model on prediction of the peritonitis treatment failure in peritonitis episodes without fungal infection or in initial peritonitis.**

| Values | P | OR | 95% CI |
| --- | --- | --- | --- |
| **Peritonitis without fungal infection (n=303)** |  |  |  |
| High density lipoprotein | .024 | .418 | 0.196 - 0.894 |
| Fibrinogen | .049 | 1.276 | 1.001 – 1.626 |
| PD duration/year | .002 | 1.019 | 1.007 – 1.031 |
| Intestinal obstruction | .006 | 5.318 | 1.6 – 17.672 |
| Diabetes mellitus | .017 | 2.484 | 1.175 – 5.251 |
| **Initial peritonitis *(n=202)** |  |  |  |
| PD duration/year | .004 | 1.017 | 1.008 – 1.046 |
| Intestinal obstruction | .019 | 8.078 | 1.413 – 46.186 |
| Diabetes mellitus | .009 | 4.745 | 1.47 – 15.317 |
| History of hemodialysis | .001 | 6.659 | 2.149 – 20.636 |

Abbreviations: OR, odds ratio. CI: confidence intervals; *:fungal peritonitis were excluded.

**Supplemental Table 3. Sensitivity analysis of predicted risk score.**

| Type of sensitivity analysis | Episodes  (N) | C statistic  (95% CI) | P for Hosmer-  Lemeshow statistic |
| --- | --- | --- | --- |
| All episodes (without fungus peritonitis) | 303 | 0.80(0.74-0.86) | 0.641 |
| Excluding patients with age >65 | 246 | 0.77(0.63-0.91) | 0.702 |
| Excluding episodes with albumin>38 | 266 | 0.77(0.71-0.84) | 0.76 |
| Excluding episodes with culture negative | 130 | 0.77(0.69-0.86) | 0.391 |
| Excluding episodes with intestinal obstruction | 290 | 0.79(0.72-0.86) | 0.713 |
| Initial episodes | 202 | 0.82 (0.73-0.90) | 0.641 |

Note: fungal peritonitis is excluded.
